# Mechanistic Evaluation and Translational Signature of Gemcitabine-induced Chemoresistance by Quantitative Phosphoproteomics Analysis with iTRAQ Labeling Mass Spectrometry

**DOI:** 10.1038/s41598-017-13330-2

**Published:** 2017-10-10

**Authors:** Qingke Duan, Hengqiang Zhao, Zhengle Zhang, Hehe Li, Heshui Wu, Qiang Shen, Chunyou Wang, Tao Yin

**Affiliations:** 1 Department of Pancreatic Surgery, Union Hospital, Tongji Medical College, Huazhong University of Science and Technology, Wuhan 430022, China; 2 Department of Clinical Cancer Prevention, The University of Texas MD Anderson Cancer Center, Houston, TX 77030 USA

## Abstract

One of the main causations of the poor prognosis of pancreatic cancer is the lack of effective chemotherapies. Gemcitabine is a widely used chemotherapeutic drug, but limited therapeutic efficacy is achieved due to chemoresistance. Recent studies demonstrated that the presence of cancer stem cells may lead to the failure of chemotherapy. Moreover, gemcitabine can promote the stemness of pancreatic cancer cells. We detected the alterations in protein phosphorylation and signaling pathways in pancreatic cancer cells after gemcitabine treatment using iTRAQ labeling LC-MS/MS, because it was featured with the advantages of strong separation ability and analysis range. A total of 232 differentially expressed phosphorylated proteins were identified in this study. Gene Ontology analysis revealed that nuclear lumen, nuclear part and organelle lumen were enriched for cell components and protein binding, poly (A) RNA binding and RNA binding were enriched for molecular function. A variety of signaling pathways were enriched based on KEGG analysis. AMPK, mTOR and PI3K/Akt pathways were verified after gemcitabine exposure. Moreover, we found there were complex interactions of phosphorylated proteins in modulating cancer stemness induced by gemcitabine exposure based on PPIs map. Our experiments may identify potential targets and strategies for sensitizing pancreatic cancer cells to gemcitabine.

## Introduction

Pancreatic cancer is a highly malignant tumor of digestive system and its incidence is increasing rapidly in recent years^1^. For the majority patients suffering from pancreatic cancer, the 5-year survival rate is less than 5% and cytotoxic treatment is the important therapeutic option and promise^2,3^. As the first-line chemotherapeutic drug, gemcitabine plays an important role in the treatment of pancreatic cancer. However, the failure of gemcitabine therapy is still a major challenge for clinicians, owing to the intrinsic or acquired resistance^4,5^. Unfortunately, limited progresses were made in improving the chemosensitivity of pancreatic cancer patients until now. In recent years, accumulated evidences demonstrate that pancreatic cancer harbors a subpopulation of cancer stem cells (CSCs), which possess strong ability of chemoresistance and tumorigenicity and may be an important causation of failure in pancreatic cancer chemotherapies^6–8^. More recently, we and others all have documented that chemotherapy can promote the stemness of pancreatic cancer cells^9–11^. However, the underlying mechanisms need to be further unraveled. Thus, elucidation of the mechanisms by which pancreatic cancer cells develop acquired stemness after gemcitabine treatment, will facilitate the identification of windows and strategies to augment the efficacy of chemotherapy in pancreatic cancer patients.

Protein phosphorylation, which is dynamic post-translational modification, can modify the protein function^12^. The phosphorylation and dephosphorylation of proteins mediated by kinases are essential events in the process of cellular signaling transduction, regulating numerous cellular processes, including cell cycle, apoptosis, proliferation, enzymatic activity, chemoresistance and survival of tumor cells^13–15^. Moreover, dynamic regulation of protein phosphorylation is vital to the signaling networks that regulate the cancer stemness^16,17^. Protein phosphorylation can be related with the activation or inhibition of specific kinases and thus classified into different signaling pathways. It was also known that phosphorylated proteins had specific molecular functions and some signaling pathways were differentially regulated through using reversible phosphorylation status of their constituent proteins^18^. Conversely, this information could be used to explain how the kinase network contributed to the different phenotypic characteristics of tumors, such as metastatic potential, stemness and chemosensitivity.

It has been reported that gemcitabine could regulate phosphorylation of multiple proteins and signaling pathways such as HAb18G/CD147-EGFR-pSTAT3 signaling pathways^19^, Nox/ROS/NF-κB/STAT3 signaling^9^, EebB2^20^, MRP-2^21^ *et al*., which were involved in regulating cancer stemness and chemoresistance. However, the comprehensive role of phosphorylated proteins and signaling pathways has not been elucidated in acquired stemness of pancreatic cancer cells after gemcitabine treatment. The isobaric tag for relative and absolute quantitation (iTRAQ) is a powerful technique which allows identification of multiple proteins and provides reliable quantitative proteome information^22^. Moreover, the iTRAQ-based quantitative phosphoproteomic analysis has multiple advantages, such as strong separation ability; labeling multiple samples and detecting large amounts of proteins at a time; high sensitivity; reliable qualitative analysis results and high level of automation^23^. Differential phosphoprotein analysis using iTRAQ technology can provide clues of up- or down-regulated phosphorylated proteins or kinases that help us predict potential signaling processes^24^. The purpose of this study was to investigate the mechanisms underlying the acquired stemness in pancreatic cancer cells after gemcitabine treatment by detecting the phosphorylated proteins using iTRAQ analysis. In this study, the Patu8988 pancreatic cancer cells that showed stem cell properties after gemcitabine exposure were analyzed with the iTRAQ quantitative analysis to characterize the changes of phosphorylated proteins. The identified phosphorylated proteins were further analyzed using KEGG (Kyoto Encyclopedia of Genes and Genomes) and GO (Gene Ontology) analyses to detect the potential signaling pathways.

In this study, we identified a total of 232 differentially expressed phosphorylated proteins. Moreover, the KEGG analysis found that a variety of signaling pathways such as AMPK, mTOR and PI3K/Akt, which may regulate the stemness of pancreatic cancer cells, were enriched after gemcitabine exposure. These results elucidated the potential mechanisms of acquired stemness in pancreatic cancer cells after gemcitabine treatment and determined potential targets for future intervention to improve the chemosensivity and prognosis of pancreatic cancer patients.

## Results

### Gemcitabine treatment promotes the stemness markers of pancreatic cancer cells

**Figure 1:**
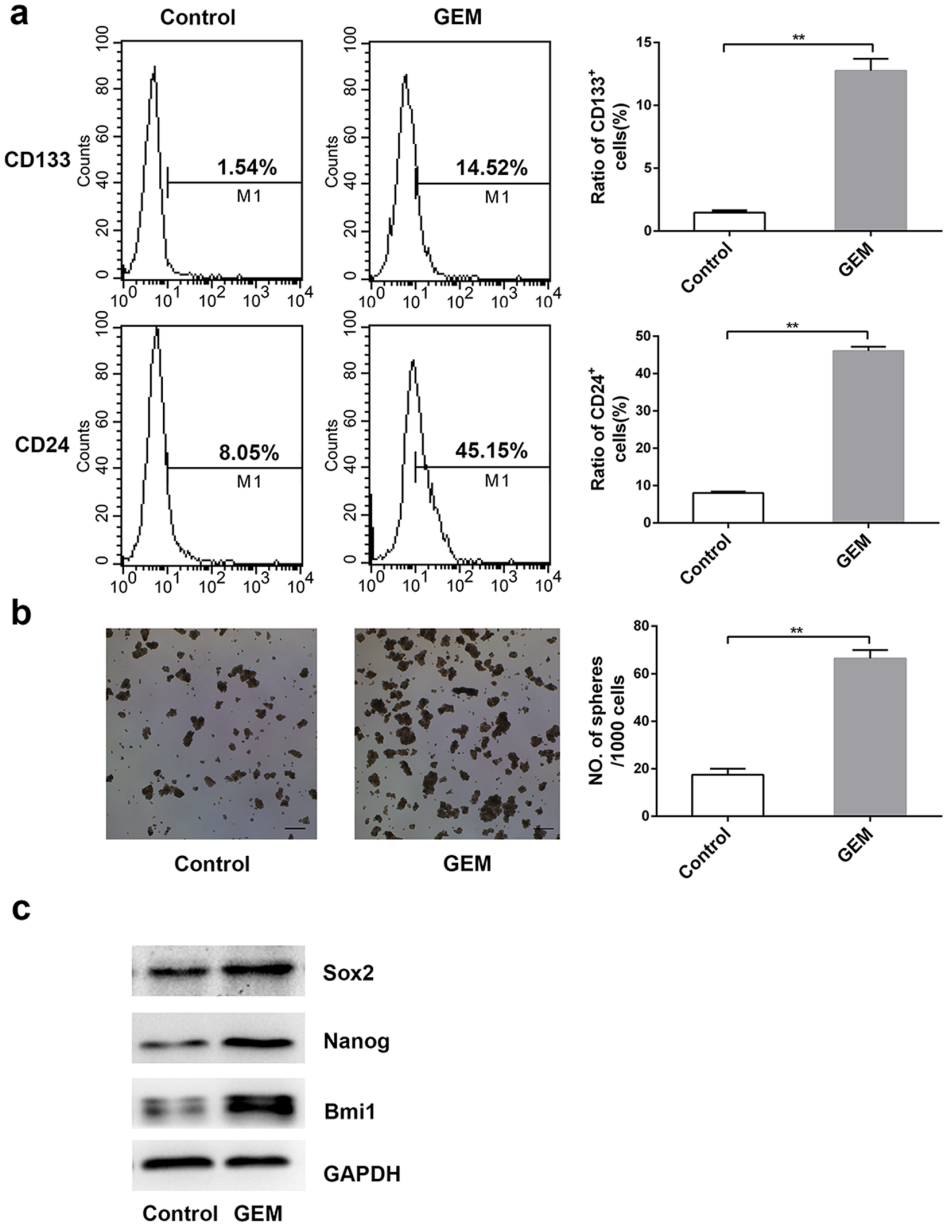
Gemcitabine treatment promotes the stemness markers of pancreatic cancer cells. (**a)** The changes of CD133+ and CD24+ CSCs treated as indicated above were measured with the use of flow cytometry. The numerical values represent the percentage of positive cells. (**b)** After gemcitabine treated for 48 h, the Patu8988 cells were subjected to the stem cell medium according to the tumor sphere-forming assay. The results were representative of three independent experiments. (**c)** The expression levels of Bmi1, Nanog and Sox2 were compared with western blot analysis after 2.5 μg/mL gemcitabine treatment for 48 h. Scale bar, 200 μm. *P < 0.05; **P < 0.01.

In this study, the Patu8988 pancreatic cancer cells were given 2.5 μg/mL gemcitabine treatment for 48 h, then pancreatic CSCs markers CD24 and CD133, were detected by flow cytometry. As shown in Fig. 1a, gemcitabine treatment increased the fractions of CD133+ cells and CD24+ cells. We next performed stem sphere formation assay, which is an important representative feature of CSCs^25^. As shown in Fig. 1b, low dose gemcitabine-treated cells showed increased ability to form stem cell spheres compared with controls. We next examined the changes of Bmi1, Nanog and Sox2 with western blot analysis, which were key stemness-associated factors^26–28^. As a result, these factors were all up-regulated after gemcitabine exposure (Fig. 1c).

### Differentially expressed phosphorylated proteins identified by iTRAQ based phosphoproteomics analysis after gemcitabine treatment

**Figure 2:**
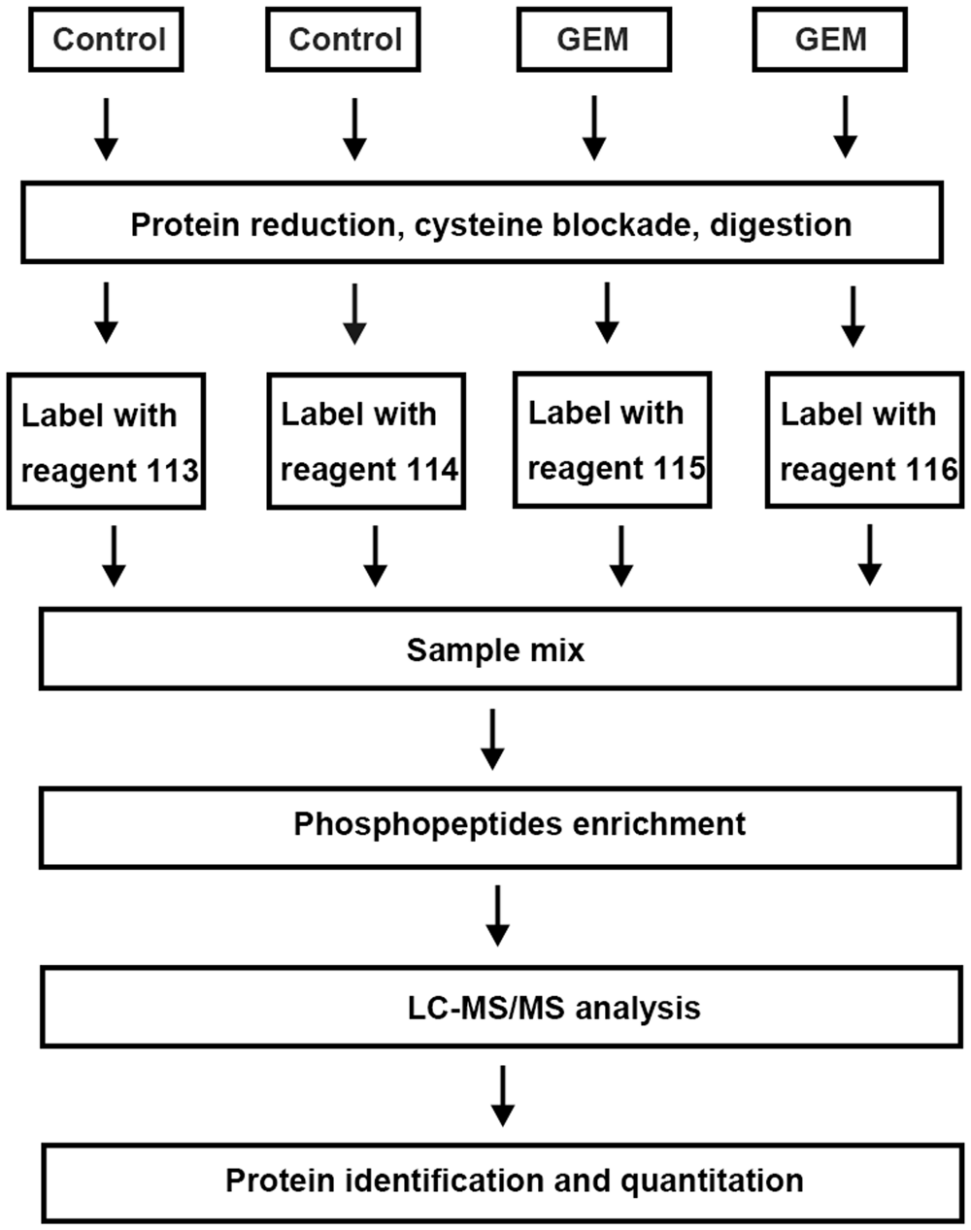
Workflow of iTRAQ labeling LC-MS/MS. The workflow of LC-MS/MS to identify differentially expressed phosphorylated proteins after gemcitabine treatment in pancreatic cancer cells.

The phosphatases and kinases occupy an important position in the regulation of signaling networks. We therefore used the quantitative phosphoproteomic technology by eight-plex iTRAQ to clarify the underlying mechanisms in our study and the workflow of iTRAQ-based phosphoproteomics analysis was shown in Fig. 2. What’s more, the representative iTRAQ mass spectra could be found as Supplementary Fig. S1.

**Figure 3:**
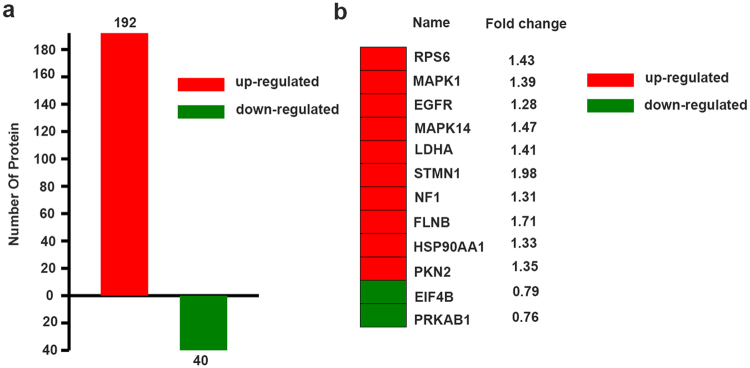
Differentially expressed phosphorylated proteins identified by iTRAQ after gemcitabine treatment. (**a**) Compared to the control group, the number of up-regulated and down-regulated phosphorylated proteins were shown in the bar graph after gemcitabine treatment. (**b**) Some differentially expressed phosphorylated proteins. Red stands for up-regulated proteins and green stands for down-regulated proteins.

Our results suggested that low dose gemcitabine treatment can promote stemness of pancreatic cancer cells. Using 0.8-fold cutoff for hypophosphorylation and 1.2-fold cutoff for hyperphosphorylation events, a total of 1785 differentially expressed phosphorylated peptides were identified in this study and 232 phosphorylated proteins were differentially expressed between the two groups among all the identified proteins. 192 phosphorylated proteins were up-regulated and the remaining 40 phosphorylated proteins were down-regulated (Fig. 3a). According to the fold change (FC) of phosphorylated proteins, we list some differentially expressed phosphorylated proteins which were discussed in our study as representative (Fig. 3b). It represented the changed phosphorylated proteins after gemcitabine exposure. What’s more, all the differentially expressed phosphorylated proteins and the expression changes could be found as Supplementary Table S1.

### GO analysis of differentially expressed phosphorylated proteins

**Figure 4:**
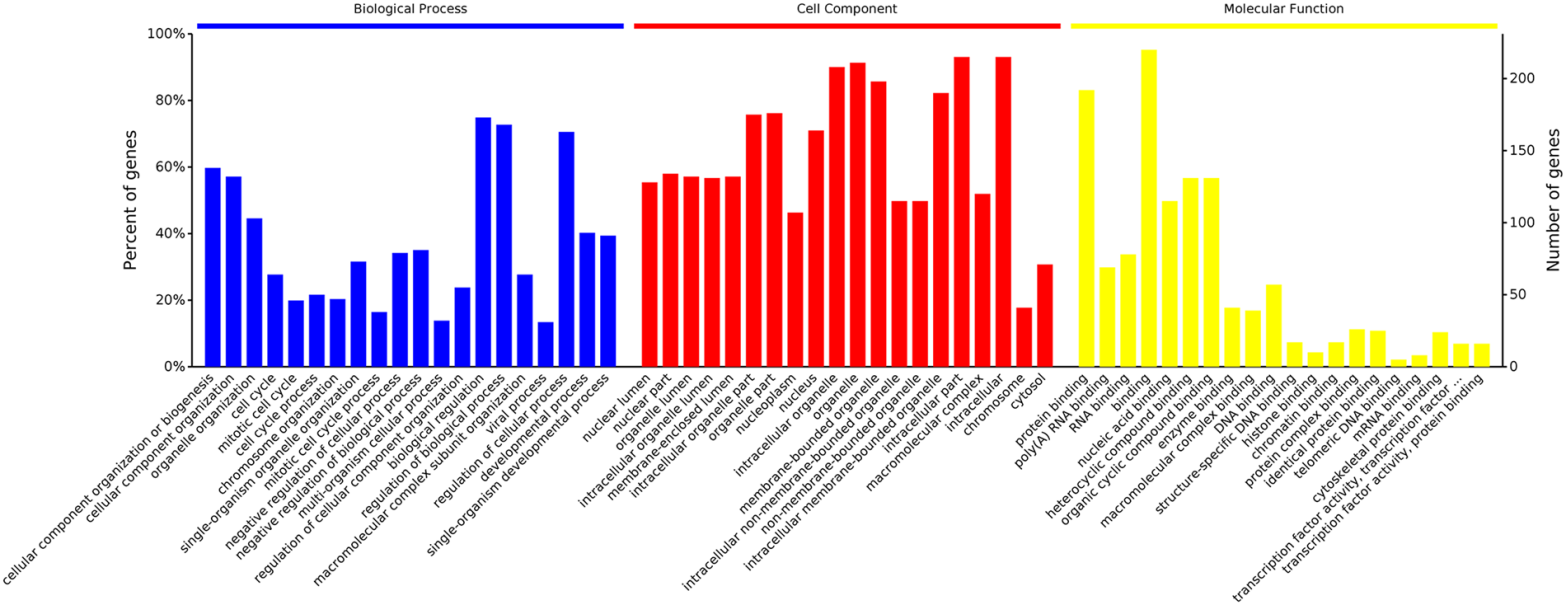
GO analysis of differentially expressed phosphorylated proteins. This graph showed the first 20 items in the enrichment results of three kinds of basic categories. The horizontal coordinate was the process name, and the vertical coordinate was the percentage of the total number of proteins that were enriched.

To ascertain how the differentially expressed phosphorylated proteins promoted stemness phenotype in pancreatic cancer cells after gemcitabine treatment, we performed GO analysis. The differentially expressed phosphorylated proteins were classified according to the biological process (BP), cell components (CC) and molecular function (MF), and the entries with significant values were identified. As shown in Fig. 4, the altered phosphorylated proteins after gemcitabine exposure mainly distributed in biological regulation, regulation of cellular process, cellular component organization or biogenesis, according to BP. Gemcitabine treatment also affected the localization of differentially expressed phosphorylated proteins to intracellular organelle, nucleoplasm, organelle and membrane-enclosed lumen, according to CC. We also found that the differentially expressed phosphorylated proteins were involved in a series of MF, including protein binding, RNA binding, nucleic acid binding, heterocyclic compound binding or organic cyclic compound binding, etc. What’s more, the percentage of associated genes in data analysis according to BP, CC and MF could be found as Supplementary Fig. S2. The most significantly and differentially expressed phosphorylated proteins were located in nuclear lumen and their function was protein binding. For example, up-regulated protein p-MAPK1 were mainly distributed in nucleus, protein binding function, cellular component organization or biogenesis process.

The same protein can be enriched into different GO items, for example the important protein EGFR. EGFR can be seen in nuclear part, organelle lumen, membrane-enclosed lumen, intracellular organelle part, organelle part. The major functions were protein binding, nucleic acid binding, heterocyclic compound binding, organic cyclic compound binding, enzyme binding. Also, the cell localization of the down-regulated protein p-AMPK were nucleus, intracellular organelle, organelle, membrane-bounded organelle, intracellular membrane-bounded organelle. The major functions were protein binding, binding and enzyme binding. The protein binding function indicated that the differentially expressed phosphorylated proteins functioned by means of interaction.

### KEGG pathway enrichment analysis of differentially expressed phosphorylated proteins

Since we observed bioinformatics analysis of differentially expressed phosphorylated proteins based on BP, CC and MF, we next performed KEGG analysis for potential signaling alterations upon gemcitabine exposure. A total of 170 KEGG pathways were enriched after gemcitabine exposure in our study. As shown in Table 1, multiple pathways associated with aggressive biological behaviors in pancreatic cancer were enriched, including FoxO signaling pathway, mTOR signaling pathway, PI3K-Akt signaling pathway, AMPK signaling pathway, MAPK signaling pathway, Ras signaling pathway, HIF-1a signaling pathway and others. What’s more, the first 10 signaling pathways could be found as Supplementary Table S2.

**Table 1:** Enrichment of differentially expressed phosphorylated proteins and signaling pathways associated with aggressive biological behaviors after gemcitabine treatment.

Pathway Name	Pathway ID	Genes
mTOR signaling pathway	hsa04150	*EIF4B, RPS6, MAPK1*
HIF-1 signaling pathway	hsa04066	*EGFR, RPS6, MAPK1, LDHA*
MAPK signaling pathway	hsa04010	*STMN1, EGFR, MAPK14, MAPK1, NF1, FLNB*
VEGF signaling pathway	hsa04370	*MAPK14, MAPK1*
PI3K-Akt signaling pathway	hsa04151	*HSP90AA1, EGFR, EIF4B, PKN2, RPS6, MAPK1*
AMPK signaling pathway	hsa04152	*PRKAB1*
Ras signaling pathway	hsa04014	*EGFR, RIN1, MAPK1, NF1*
FoxO signaling pathway	hsa04068	*EGFR, SKP2, PRKAB1, USP7, MAPK14, MAPK1*
NOD-like receptor signaling pathway	hsa04621	*HSP90AA1, ERBB2IP, MAPK14, MAPK1*
Central carbon metabolism in cancer	hsa05230	*EGFR, MAPK1, LDHA*
Signaling pathways regulating pluripotency of stem cells	hsa04550	*MAPK14, MAPK1*

### Effects of targeted anticancer agents on Patu8988 cells

**Figure 5:**
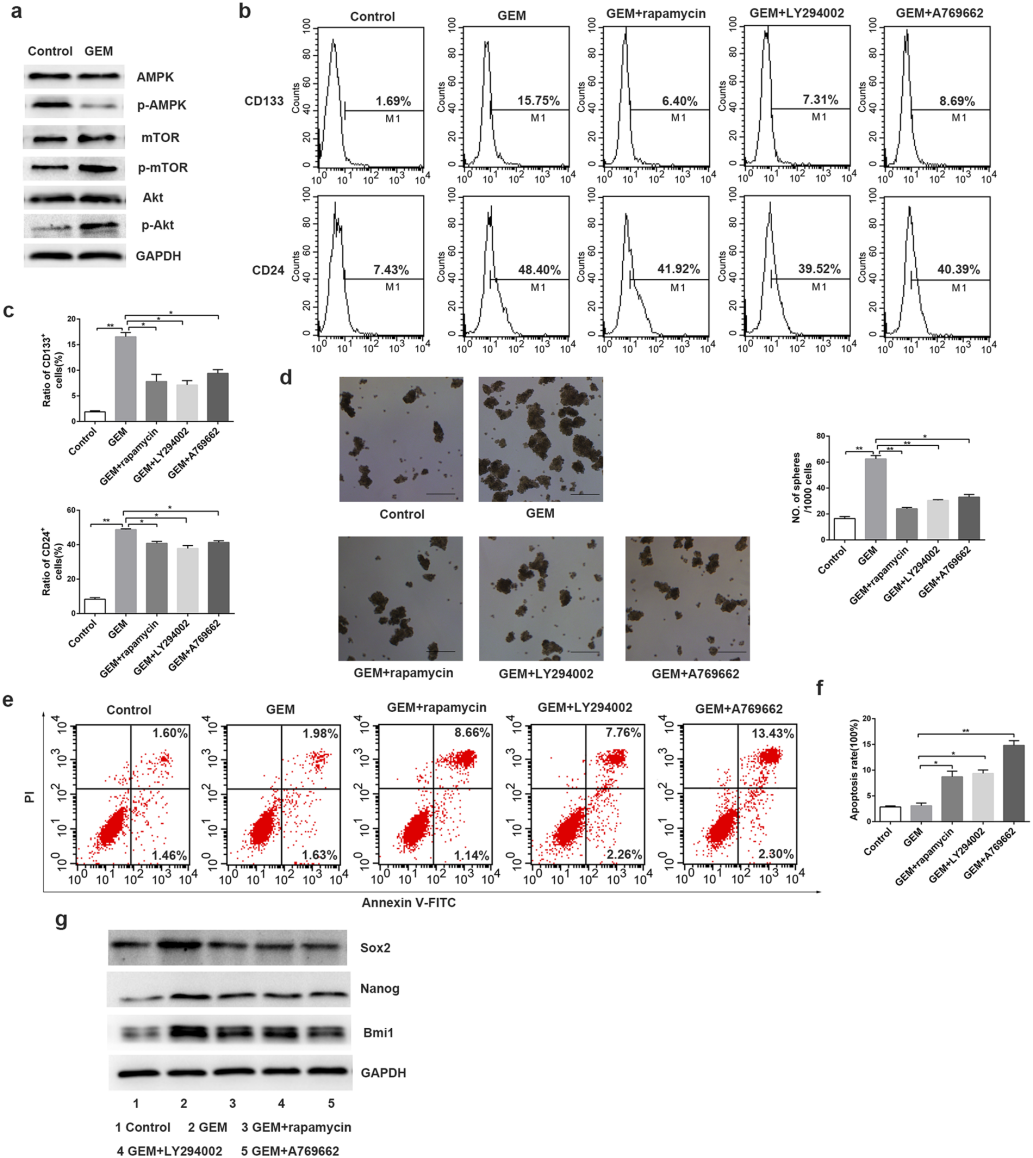
Effects of targeted anticancer agents on Patu8988 cells. (**a**) Patu8988 cells were treated with gemcitabine for 48 h at the concentrations of 2.5 μg/mL, and the expression of phosphorylated proteins were detected by Western Blot. (**b**,**c**) The pancreatic CSCs markers CD133-PE, CD24-FITC after gemcitabine and the corresponding inhibitors/activator treatment were measured by flow cytometry. The cells were treatment with gemcitabine (2.5 μg/mL, 48 h), rapamycin (1 μM, 48 h), LY294002 (20 μM, 24 h) and A-769662(20 μM, 2 h). The numerical values represent the percentage of positive cells. (**d**) After inhibitors or activator treatment, the Patu8988 cells were performed with the tumor sphere-forming experiments in stem cell medium. (**e,f**) Dot-plots represented the apoptotic status of Patu8988 cells using Annexin V-FITC/PI method. The dot-plots in the upper right and lower right quadrant were considered as the percentage of apoptotic cells. (**g**) The expression levels of Bmi1, Nanog and Sox2 were compared with western blot analysis after gemcitabine and the corresponding inhibitors/activator combination treatment. The results were representative of three independent experiments. Scale bar, 200 μm. *P < 0.05; **P < 0.01.

To verify that abnormal activation of the enriched signaling pathways induced by gemcitabine could confer stemness of pancreatic cancer cells, we first detected the expression of selected phosphorylated proteins identified by iTRAQ with Western Blot analysis. The results showed that the expression of p-Akt and p-mTOR increased, and the expression of p-AMPK decreased after gemcitabine treatment (Fig. 5a). The results of western blot further verified that changes of these signaling pathways were involved in the acquired CSCs phenotypes after gemcitabine treatment.

To further verify the role of these changed phosphorylated proteins involved in the acquired stemness of pancreatic cancer after gemcitabine treatment, selected kinase inhibitors or activator, including rapamycin (mTOR inhibitor), LY294002 (PI3K/Akt inhibitor) and A769662 (AMPK activator) combined with gemcitabine were treated on pancreatic cancer cells. The pancreatic CSCs markers, CD24 and CD133, were detected using flow cytometry in the experiments. As shown in Fig. 5b,c, the ratios of CD133+ cells and CD24+ cells all decreased compared with gemcitabine treatment alone. After treated with these targeted anticancer agents, the spheres formation decreased too (Fig. 5d). We further detected the cytotoxic effect of gemcitabine combined with targeted anticancer agents on pancreatic cancer cells using apoptosis detection assay. The flow cytometer analysis showed that the apoptotic cells were increased after combination treatment compared with gemcitabine treatment alone (Fig. 5e,f). We next examined the expression level changes of Bmi1, Nanog and Sox2 after gemcitabine treatment in pancreatic cancer cells with western blot analysis. As shown in Fig. 5g, they were all down-regulated after combination treatment. These results suggest that modulation of mTOR, PI3K/Akt or AMPK signaling pathway with kinase inhibitors or activator is an effective strategy to block stemness and increase the chemosensitity of pancreatic cancer cells.

### Interaction of differentially phosphorylated proteins and signaling pathways

**Figure 6:**
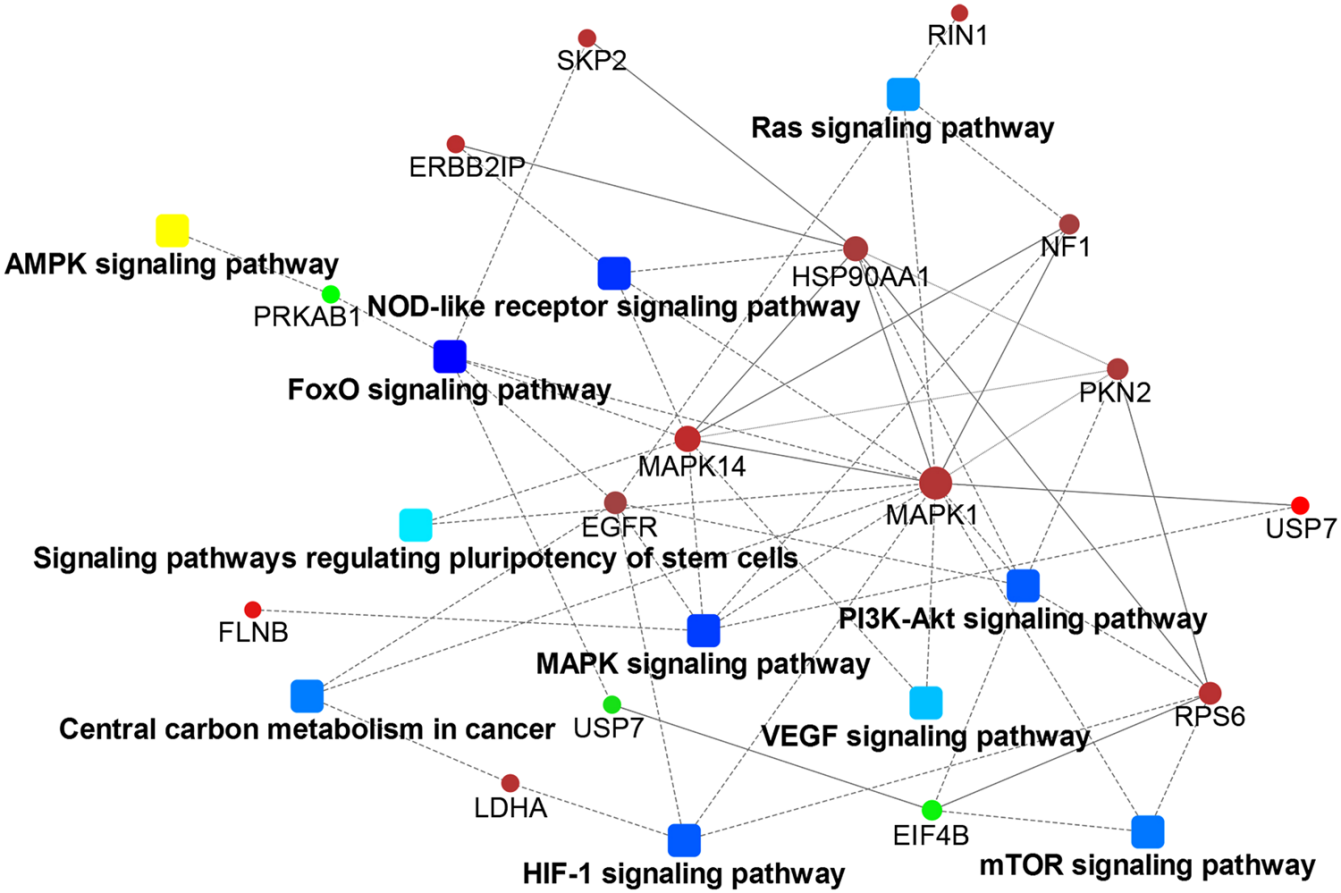
Interaction of different phosphorylated proteins. Biological network among phosphorylated proteins were shown by lines, which represented the interactions.

The protein-protein interactions (PPIs) map in cells can help us better understand the biological and molecular mechanisms of cancer biological behavior comprehensively^29^. We thus further investigated whether these phosphorylated proteins were interacted with each other based on the results of iTRAQ. As shown in Fig. 6, a large number of phosphorylated proteins such as EGFR, MAPK1, HSP90AA1 and RPS6 showed close relationship with other phosphorylated proteins after gemcitabine treatment. The same phosphorylated protein could be enriched into different signaling pathways, for example the phosphorylated EGFR could be enriched in pathways including HIF-1, MAPK, PI3K/Akt, Ras, FoxO and Central carbon metabolism in cancer. The corresponding signaling pathways in which these proteins were located may interact with each other as well. In summary, these results showed evidence for the involvement of phosphorylated proteins in modulating PPIs induced by gemcitabine exposure.

## Discussion

Our studies and others revealed that low dose gemcitabine treatment could induce the CSC phenotype and tumor sphere-forming ability in pancreatic cancer cells^9,30^. This process is related with increased tumorigenesis, chemoresistance and metastasis of pancreatic cancer, which may be an underlying mechanism of resistance to gemcitabine. So, elucidating the mechanisms that govern the acquired stemness in pancreatic cancer after gemcitabine treatment may be helpful to sensitize the chemotherapy of pancreatic cancer.

The differences of proteins and pathways between Panc1 CSCs and Panc1 parental cells has been reported by iTRAQ-based proteomic analysis. It suggested that mevalonate pathways and fatty acid synthesis played an important role in ensuring viability of pancreatic cancer stem cells^31^. In our study, we identified the phosphorylated proteins and signaling pathways relevant to the acquired stemness induced by low dose gemcitabine in pancreatic cancer cells by LC-MS/MS. The GO results showed the biological process, cell localization and molecular function of up-regulated or down-regulated phosphorylated proteins. The identified phosphorylated proteins functioned primarily by binding to other proteins or RNAs to regulate various biological processes in cells, inducing CSCs characteristics. Our iTRAQ results showed that a total of 232 phosphorylated proteins were significantly altered and we also found multiple activated signaling pathways involved in the acquired stemness of pancreatic cancer cells after gemcitabine treatment with KEGG analysis. In these altered signaling pathways, we focused on the study of AMPK, Akt and mTOR as representatives, which have been recognized as associated with malignant behaviors of pancreatic cancer.

The iTRAQ results of our study showed that the expression of p-AMPK decreased after gemcitabine treatment, which was verified in subsequent Western Blot analysis. Moreover, when treated with AMPK activator combined with gemcitabine, the stemness of pancreatic cancer including stem cell markers and sphere-forming capability decreased. This suggested that inhibition of AMPK signaling pathway was involved in promoting stemness in pancreatic cancer cells treated with gemcitabine. Combined with existing report that AMPK activation increased the chemotherapy sensitivity of pancreatic cancer cells to gemcitabine^32^, we concluded that gemcitabine promoted the stemness properties by inhibiting AMPK signaling pathway in pancreatic cancer cells. Previous literature reported that p-AMPK increased slightly but not significantly in AsPC-1 pancreatic cancer cells after 300 nM gemcitabine treatment^33^, which may result from a relatively high concentration. Our results also verified that the activity of PI3K/Akt and mTOR signaling pathways increased after gemcitabine treatment, and the stemness of pancreatic cancer decreased after treated with PI3K/Akt and mTOR inhibitors combined with gemcitabine respectively. This was consistent with the reports that inhibition of PI3K/Akt or mTOR signaling pathways could enhance the chemotherapeutic efficacy of gemcitabine on pancreatic cancer patients^34,35^. These findings suggest that activation of PI3K/Akt and mTOR signaling pathways may participate in promoting stemness of pancreatic cancer treated with gemcitabine.

Our results also demonstrated that multiple signaling pathways and phosphorylated proteins were altered in Patu8988 cells after treated with low dose gemcitabine, such as MAPK, HIF, FoxO and others. MAPK can be divided into 4 subfamilies, ERK, p38, JNK, ERK5. In our study, the expression of phosphorylated ERK increased after gemcitabine treatment (fold change 1.39) by LC-MS/MS, which indicated the activation of MAPK signaling pathway. Previous studies verified that, CSC surface markers, such as CD44 and CD133, could be reduced when ERK signaling pathway was inhibited^36,37^. MAPK pathway may be involved in the acquired stemness in pancreatic cancer cells treated with gemcitabine in our study.

In this study, we also found the up-regulation of EGFR, p38 and SKP2, which were enriched into FoxO signaling pathway activation after gemcitabine exposure. It has been reported that the p38 could phosphorylate FoxO directly, while the p-Fox0 will be exported into cytoplasm, degraded and lose the transcriptional activity^38^. Skp2 could suppress tumor-suppressor effects of FoxO through promoting the degradation of FoxO in the nucleus via ubiquitination^39^. The decreased nuclear accumulation of FoxO will promote proliferation of cells and CSCs phenotype, and is associated with poor prognosis by blocking the downstream signaling pathways^40,41^. Our KEGG analysis also found activation of HIF signaling pathway after low dose gemcitabine treatment. Studies have demonstrated that overexpression of HIF is associated with poor prognosis in patients with pancreatic cancer^42^. The activation of HIF signaling pathway could promote the chemoresistance and is critical in maintaining the characteristics of CSCs in pancreatic cancer^43–45^. HIF signaling pathway may be involved in the acquired stemness of patu8988 cells treated with low dose gemcitabine.

EGFR is a receptor tyrosine kinase, which is involved in the occurrence and development of a variety of malignant tumors, such as breast cancer^46^, non-small cell lung cancer^47^ and pancreatic ductal adenocarcinoma^48^. In human pancreatic cancer, the expression level of EGFR is closely related to the prognosis of the disease^49^. Moreover, EGFR was reported to modulate the side population in human carcinoma cell lines that possessed stem cell properties^50^. Our results showed that phosphorylated EGFR increased (fold change 1.28) with the increase of stemness of pancreatic cancer cells treated with gemcitabine. Moreover, our results also suggest a central position of EGFR in regulating and maintaining CSCs induced by gemcitabine because our PPI analysis showed that EGFR was interacted with multiple signaling pathways.

In our study, we achieved the phosphorylation changes of multiple proteins by quantitative phosphoproteomics analysis. But these phosphorylated proteins may not function alone, as they have to interact with others to establish networks of signaling pathways and complete a series of regulatory functions. Networks of PPIs based on the string database have proved to be a tremendous vehicle to develop new hypotheses, design novel experiments and analyze proteomic data. From our PPIs results, there are potential synergistic effects among different signaling pathways. More than 90% of pancreatic cancer patients have a K-Ras mutation and the activated Ras may drive the progression of pancreatic cancer by regulating the activity of multiple downstream pathways. Our KEGG results suggested that gemcitabine treatment promotes the activation of Ras. Moreover, Ras signaling pathway is interacted with multiple signaling including PI3K/Akt, MAPK, EGFR, HIF and others in our PPI analysis. Our study emphasized the pivotal role of K-Ras in acquired stemness of pancreatic cancer cells treated with low dose gemcitabine. There may be reciprocal activation of different signaling pathways among them.

Our results also showed the activation of signaling pathways involved in carbon metabolism after gemcitabine treatment, which interacted with changes of multiple signaling pathways including PI3K/Akt, AMPK, HIF, mTOR and others in our PPI analysis. It has been reported that Akt, AMPK, HIF-1a and FoxO all play important roles in glycolysis^51–54^. Meanwhile, the metabolic reprogramming may govern the phenotypic changes of cancer stemness and the enhanced glycolysis could contribute to the self-renewal, treatment resistance and sphere-forming ability of CSCs^55,56^. Thus, we propose a hypothesis that acquired gemcitabine exposure can induce stemness in pancreatic cancer via modulating protein kinases mediated carbon metabolism.

In conclusion, our results demonstrate that low dose gemcitabine treatment can cause the changes of protein kinases, phosphatases and the corresponding signaling pathways, which may play important roles to induce stem cell characteristics in pancreatic cancer cells. There may be complicate interactions among different signaling pathways in developing such stemness in the context of gemcitabine exposure in pancreatic cancer.

## Methods

### Cell culture

The pancreatic cancer cell line Patu8988 was obtained from Keygen (Key Gen Bio Tech, China) and was cultured in DMEM supplemented with 10% FBS, 1% penicillin/streptomycin mixture at 37 °C with 5% CO2. The cells were treated with gemcitabine (2.5 μg/mL, 48 h), or combined with rapamycin (1 μM, 48 h), LY294002 (20 μM, 24 h) and A-769662 (20 μM, 2 h).

### Western blot analysis

The total proteins were extracted with RIPA lysis buffer contained protease inhibitors PMSF and cocktails. The proteins were quantified by BCA assay kit (Beyotime Biotechnology, Shanghai, China) according to the manufacturer’s protocol. Equal amounts of proteins were loaded onto 10% SDS-polyacrylamide gel electrophoresis and were transferred to PVDF membrane (Millipore, Billerica, MA, USA). The membrane was then blocked with 5% non-fat milk for 1 h and incubated with primary antibodies overnight. Primary antibodies against GAPDH (1:1000), mTOR (1:1000), p-mTOR (1:1000), Akt (1:1000), p-Akt (1:1000), AMPK (1:1000), p-AMPK (1:1000) were all purchased from CST(Cell Signaling Technology, Danvers, MA, USA). After washed with TBST, the membrane was incubated with horseradish peroxidase (HRP)-conjugated secondary antibodies and was visualized with enhanced chemiluminescence (Pierce, Thermo Fisher, Waltham, MA, USA). The GAPDH was used as internal control.

### Flow cytometry analysis

The changes in CSC (cancer stem cells) markers were detected by flow cytometry analysis. Briefly, the treated cells were collected and dissociated into single cell suspension. Each sample was given 10 μL antibody and incubated for 30 minutes at 4 °C. After washing with PBS, the samples were analyzed with flow cytometer. Antibody against CD24-FITC was obtained from BD Pharmingen (San Diego, CA, USA) and anti-CD133-PE antibody was purchased from Miltenyi Biotec (Bergisch Gladbach, Germany). Gemcitabine, mTOR inhibitor (rapamycin) and AMPK activator (A-769662) were purchased from Selleck chemicals (Selleck.cn, Shanghai, China). PI3K/Akt inhibitor (LY294002) was purchased from Beyotime. The flow cytometry analysis are determined from three independent experiments.

### Apoptosis detection

The pancreatic cancer cells were seeded into 6-well plates and received different treatment. All the cells were collected including attached and floating cells before detection. Then, they were stained with Annexin V-FITC/PI (Antgene Biotechnology, Wuhan, China) according to protocols. The apoptotic cells were detected with flow cytometer analysis.

### Protein preparation and iTRAQ labeling

In order to further clarify the underlying mechanisms, we used iTRAQ to compare the changes of phosphorylated proteins. Pancreatic cancer cells treated with DMSO or gemcitabine for 48 h were harvested, protein extracted, quantified, reduced, cysteine blocked, digested and labeled, then the phosphorylated peptides were enriched and analyzed on mass spectrometer. Concretely speaking, proteins from the Patu8988 pancreatic cancer cells after gemcitabine treatment were precipitated with cold acetone for 2 h at −20 °C, pelleted by centrifugation at 12000 g, air-dried and dissolved into dissociation buffer. The total protein solution was quantified by BCA protein assay kit and protein lysate was added to the samples of high concentration to make the final concentration the same between the two groups. What’s more, the same amount and same volumes of proteins for two groups were given reductive alkylation and enzymatic hydrolysis. Then, the samples were labeled with isobaric tagging reagents according to protocols. iTRAQ reagents were obtained from AB SCIEX. The control group was labelled with 113 and 114; the gemcitabine treated group was labelled with 115 and 116. The results were obtained from three experiments. For every experiment, two independent replicate samples of control and gemcitabine treated group were included.

### Phosphopeptides enrichment and LC-MS/MS analysis

The dry samples were re-suspended with 1 ml Nano-RPLC buffer A (0.1% formic acid, 2% acetonitrile). After adding 3 ml TiO_2_ phosphobind buffer, the phosphopeptides were enriched according to the protocols of TiO2 kits (TiO2 beads, GL Sciences, Japan). After the enrichment, the solution was freeze dried and re-dissolved in Nano-RPLC buffer A. The online Nano-RP liquid chromatography was employed on EKsigent nanoLC-Ultra^TM^ 2D system (AB SCIEX). The treated samples were loaded on C_18_ NanoLC trap column (3 cm × 100 μm, C_18_, 3 μm, 150 Å) and washed for 10 min at 2 μL/min. The analytical column was C_18_ reverse phase column (75 μm × 15 cm, C_18_, 3 μm, 120 Å, ChromXP EKsigent) and the elution gradient changed from 5% to 35% in 70 min. The Triple TOF5600 system (AB SCIEX) combined with Nanospray 3 source (AB SCIEX, USA) were used to carry out the MS data acquisition.

### Identification and quantification of phosphorylated proteins

Data was processed and analyzed according to the Protein Pilot Software v.5.0 (AB SCIEX, USA) software. Match the experimental data with the theory data of mass spectrometry in the database to get the results of the identification. Quantification of phosphorylated proteins is based on the signal strength of each peptide segment labeled by iTRAQ. The phosphorylated proteins are qualitatively defined based on the comparison between the amino acid sequence of peptide segments and the peptide information in the database. The changes in differential phosphorylated proteins are determined according to the set difference times. The databases include David, Quick GO, String, Gene Ontology, Kyoto Encyclopedia of Genes and Genomes. We use KEGG to map the differential phosphorylated proteins to model organisms and different phosphorylated proteins are enriched to different pathways. Then, we identify the signaling pathways involved in differentially phosphorylated proteins and their functions through the KEGG visualization pathway.

### Statistical analysis

The data are expressed as mean ± SEM. The Western Blot results were analyzed by Image Lab 3.0 software (Bio-Rad, Hercules, CA, USA). The Student’s *t* test was performed to compare and analyze between two groups. The statistical analysis was evaluated using SPSS 21.0 software. P < 0.05 was considered to be statistically different.

### Data Availability

The datasets generated and analyzed during the current study are available from the corresponding author on reasonable request.

## Electronic supplementary material

Mechanistic Evaluation and Translational Signature of Gemcitabine-induced Chemoresistance by Quantitative Phosphoproteomics Analysis with iTRAQ Labeling Mass Spectrometry
