# Supplementary material for: Mechanistic Evaluation and Translational Signature of Gemcitabine-induced Chemoresistance by Quantitative Phosphoproteomics Analysis with iTRAQ Labeling Mass Spectrometry
Source: Sci Rep. 2017 Oct 10;7:12891. doi: 10.1038/s41598-017-13330-2 (PMC5634998; doi:10.1038/s41598-017-13330-2)
Supplement: Supplementary file 1 — Mechanistic Evaluation and Translational Signature of Gemcitabine-induced Chemoresistance by Quantitative Phosphoproteomics Analysis with iTRAQ Labeling Mass Spectrometry [file 41598_2017_13330_MOESM1_ESM.pdf]

Mechanistic Evaluation and Translational Signature of Gemcitabine-induced Chemoresistance by Quantitative Phosphoproteomics Analysis with iTRAQ Labeling Mass Spectrometry

Qingke Duan[#,1], Hengqiang Zhao[#,1], Zhengle Zhang[1], Hehe Li[1], Heshui Wu[1], Qiang Shen[2], Chunyou Wang[1], Tao Yin[*,1]

Supplementary Figure S1

The representative iTRAQ mass spectrum (bottom spectrum) and reporter ion region (top spectrum) for the peptide of p-MAPK1, which was up-regulated after gemcitabine treatment.

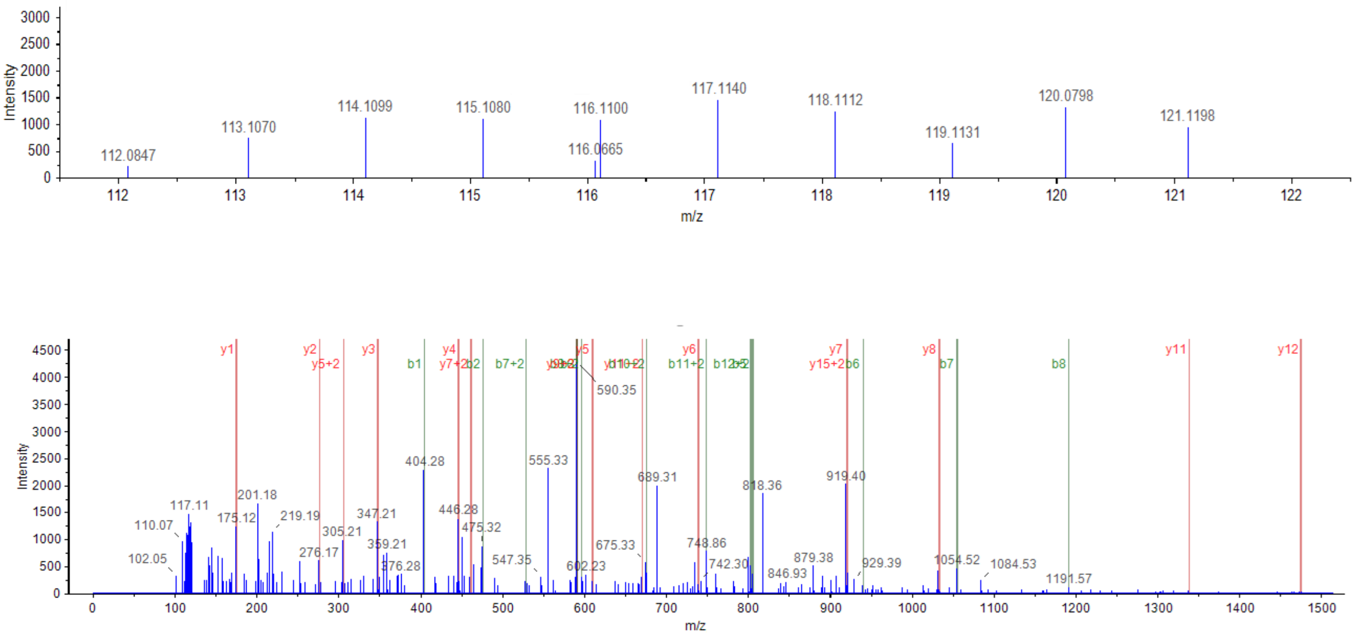

Supplementary Figure S2

Biological process, Cell components and Molecular function results of differentially expressed phosphorylated proteins after gemcitabine treatment.

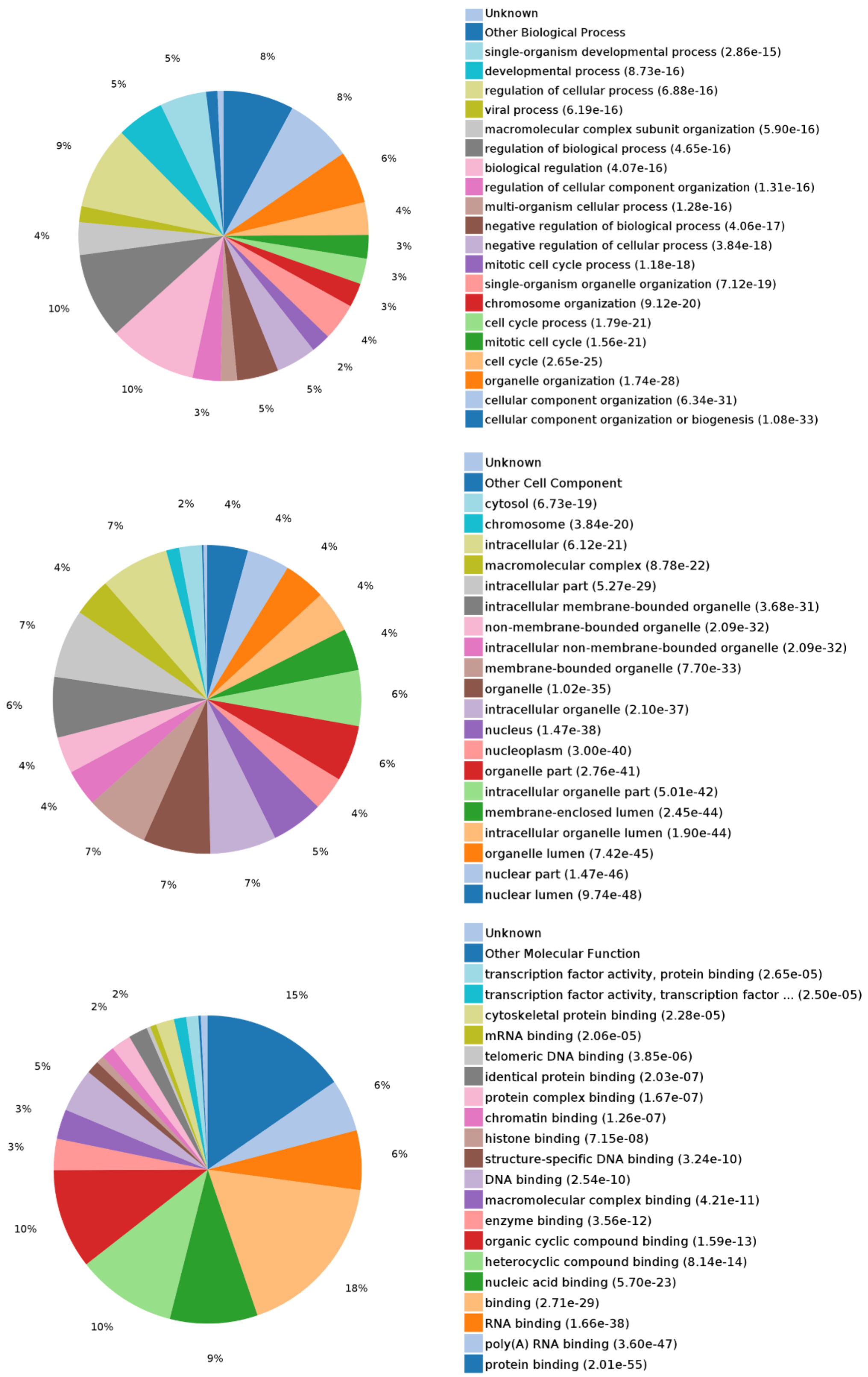

Supplementary Table S1

All the differentially expressed phosphorylated proteins and the expression changes after gemcitabine treatment. Fold change < 0.8 represents down-regulated protein and > 1.2 represents up-regulated protein.

**Gene**	**Fold change**	**Gene**	**Fold change**	**Gene**	**Fold change**	**Gene**	**Fold change**
RB1	1.21	*HIST1H4A*	0.52	*USP39*	1.23	*RRM2*	1.82
HDAC1	1.23	*HSP90AA1*	1.33	*PDIA6*	1.35	*TAF1*	1.44
SMC3	1.34	*ERBB2IP*	1.45	*HNRNPU*	2.22	*NF1*	1.31
MCM6	5.72	*PABPN1*	2.34	*TPI1*	1.92	*EIF2S2*	1.24
SKP2	1.42	*CSTF3*	1.21	*RAD23B*	1.38	*UNG*	0.70
MCM3	0.74	*UPF1*	0.73	*LMNA*	1.23	*TROVE2*	1.22
ATR	1.39	*SYMPK*	1.56	*PCYT1A*	1.24	*SLC4A2*	1.24
PRKDC	1.34	*PAPOLA*	1.29	*HMGB1*	0.67	*FTH1*	1.42
MAD1L1	1.35	*PRKAB1*	0.76	*SSB*	1.34	*LDHA*	1.41
PTTG2	1.25	*TJP2*	1.21	*ALS2*	1.24	*SGPL1*	1.25
CDC26	1.30	*TJAP1*	1.37	*HMGN1*	1.22	*NUP155*	0.56
BUB1B	1.53	*STAT1*	1.22	*H2AFX*	1.96	*PPIP5K2*	1.25
RAD50	1.35	*MARCKSL1*	1.77	*PKP2*	1.37	*FKBP4*	1.31
XRCC6	1.62	*MED1*	0.61	*TLN1*	2.03	*LIMA1*	1.35
CTTN	1.39	*EIF4B*	0.79	*CYLD*	1.29	*TOP2A*	2.09
NCL	1.29	*RPS6*	1.43	*PHAX*	1.26	*CCDC86*	0.78
KRT18	1.51	*FAM21A*	1.27	*DNAJC5*	1.25	*RSF1*	1.26
TUBA1C	1.42	*RAB11FIP1*	1.21	*SETD2*	0.73	*TRIM28*	1.22
OCLN	1.36	*ARFGAP1*	1.36	*PSMB7*	1.58	*HDGF*	1.23
DYNC1LI1	1.34	*GBF1*	1.25	*EDC3*	0.77	*ZRANB2*	1.32
MYH10	1.24	*FAM21C*	1.24	*GPSM1*	1.53	*PPME1*	1.21
PKN2	1.35	*EHD2*	1.22	*RPL22L1*	0.72	*KRT8*	1.20
MAPK14	1.47	*CLTC*	0.70	*UAP1*	1.70	*PDS5B*	1.35
MAPK1	1.39	*UTP18*	0.68	*RIN1*	1.43	*TCEA1*	1.21
FLNB	1.71	*NOP58*	0.76	*ARHGAP5*	1.23	*ANLN*	1.28
EGFR	1.28	*RIOK1*	0.75	*RPL31*	0.64	*CTR9*	1.23
NOTCH2	1.28	*EIF6*	1.23	*KPNA2*	1.38	*HNRNPH1*	1.22
HNRNPK	1.47	*STMN1*	1.98	*HNRNPA3*	0.62	*UTP3*	1.23
USP7	0.71	*KIF23*	1.42	*PEX19*	1.24	*HMGA1*	1.61

Continued Table S1

Gene	Fold change	Gene	Fold change	Gene	Fold change	Gene	Fold change
LARP4	1.30	*RPRD1A*	1.36	*KCTD12*	1.21	*CDK3*	1.50
SPTBN1	1.38	*INCENP*	1.32	*KHSRP*	1.23	*RBM10*	1.23
PRRC2A	1.41	*HDGFRP2*	1.57	*H1FX*	0.63	*GSDMD*	1.21
IWS1	1.22	*RSL1D1*	0.79	*DDX1*	1.21	*NAP1L1*	1.55
TCEAL3	1.30	*GAPVD1*	0.46	*GATAD2B*	1.34	*BRPF1*	0.74
FNBP4	1.30	*CAMSAP2*	1.26	*TMEM40*	1.89	*NOP2*	1.24
SLTM	1.24	*TGOLN2*	2.06	*PPP1R13L*	1.22	*NCAPD3*	1.31
RTN4	1.23	*MATR3*	1.25	*GTF3C2*	1.23	*KDM5A*	1.26
PPAN	1.23	*SCFD1*	0.62	*ZCRB1*	1.75	*ANXA1*	1.30
CAAP1	1.28	*NASP*	0.73	*CEP76*	1.39	*ZBTB7A*	1.23
API5	1.34	*ZNF830*	1.25	*GPRC5A*	1.44	*NFAT5*	1.21
RBM33	1.38	*STRN*	1.21	*SERBP1*	0.62	*UBXN7*	1.34
CDCA2	1.21	*NSRP1*	1.21	*ADAMTS13*	1.52	*UFL1*	1.45
PCM1	0.74	*ZNF280C*	1.33	*PACS1*	1.21	*DNAJB6*	0.74
PEA15	1.68	*CLIC4*	1.74	*DCAF8*	1.21	*SIN3B*	2.39
USP10	1.23	*SNX24*	1.22	*CAMSAP1*	1.24	*IGSF3*	1.27
TXLNA	1.27	*PA2G4*	1.27	*SAMD4B*	0.77	*PLIN3*	1.24
CLIP1	1.41	*SLC12A4*	1.49	*RRP12*	0.78	*SPAG9*	1.61
YY1	1.26	*ZHX1*	1.21	*C17orf85*	1.21	*DNPH1*	1.40
CAST	1.54	*GTF3C4*	1.21	*CCDC88A*	1.53	*RNF113A*	1.39
BTF3	0.75	*BAZ2A*	1.40	*KIF4B*	1.76	*ZNF593*	0.79
ANXA2	1.25	*EIF3K*	1.67	*TERF2*	1.44	*A6NL46*	0.61
OXSR1	1.21	*TMOD3*	1.28	*SURF2*	0.56	*ESYT2*	1.36
PGRMC1	1.37	*TERF2IP*	1.30	*NONO*	1.26	*PTPN3*	1.24
CHAF1B	1.56	*GPN1*	0.62	*PUM1*	0.79	*SORBS3*	1.26
THUMPD1	0.63	*WDR55*	1.42	*TTLL12*	1.30	*NFIB*	1.43
PPP4R2	1.25	*SLC39A3*	1.30	*HNRNPD*	1.27	*PPP1R7*	1.37
ZNF687	0.63	*SPAG5*	1.56	*IK*	1.47	*CLASP1*	1.76
KTN1	1.26	*PPP1R10*	1.24	*TWF1*	1.34	*DEK*	0.74

Supplementary Table S2

The first 10 signaling pathways enriched by KEGG analysis after gemcitabine treatment.

Pathway Name	Pathway ID	Genes	Count
Cell cycle	hsa04110	*RB1,HDAC1,SMC3,MCM6,SKP2,MCM3,ATR,PRKDC, MAD1L1,PTTG2,CDC26,BUB1B*	12
Non-homologous end-joining	hsa03450	*PRKDC,RAD50,XRCC6*	3
Pathogenic Escherichia coli infection	hsa05130	*CTTN,NCL,KRT18,TUBA1C,OCLN*	5
Salmonella infection	hsa05132	*DYNC1LI1,MYH10,PKN2,MAPK14,MAPK1,FLNB*	6
Dorso-ventral axis formation	hsa04320	*EGFR,NOTCH2,MAPK1*	3
Progesterone-mediated oocyte maturation	hsa04914	*HSP90AA1,MAD1L1,CDC26,MAPK14,MAPK1*	5
NOD-like receptor signaling pathway	hsa04621	*HSP90AA1,ERBB2IP,MAPK14,MAPK1*	4
Viral carcinogenesis	hsa05203	*RB1,HNRNPK,HDAC1,SKP2,MAD1L1,USP7,HIST1H4A, MAPK1*	8
mRNA surveillance pathway	hsa03015	*PABPN1,CSTF3,UPF1,SYMPK,PAPOLA*	5
FoxO signaling pathway	hsa04068	*EGFR,SKP2,PRKAB1,USP7,MAPK14,MAPK1*	6
